# *Vibrio tapetis* Displays an Original Type IV Secretion System in Strains Pathogenic for Bivalve Molluscs

**DOI:** 10.3389/fmicb.2018.00227

**Published:** 2018-02-19

**Authors:** Graciela M. Dias, Adeline Bidault, Patrick Le Chevalier, Gwenaëlle Choquet, Clio Der Sarkissian, Ludovic Orlando, Claudine Medigue, Valerie Barbe, Sophie Mangenot, Cristiane C. Thompson, Fabiano L. Thompson, Annick Jacq, Vianney Pichereau, Christine Paillard

**Affiliations:** ^1^Laboratoire des Sciences de l'Environnement Marin, Université de Bretagne Occidentale, UMR 6539 UBO/Centre National de la Recherche Scientifique/IRD/Ifremer, Institut Universitaire Européen de la Mer, Plouzané, France; ^2^Laboratório de Microbiologia, Instituto de Biologia, Universidade Federal do Rio de Janeiro, Rio de Janeiro, Brazil; ^3^Laboratoire de Biotechnologie et Chimie Marine, Université de Bretagne Occidentale, Quimper, France; ^4^Centre for GeoGenetics, Natural History Museum of Denmark, University of Copenhagen, Copenhagen, Denmark; ^5^Laboratoire d'Anthropobiologie Moléculaire et d'Imagerie de Synthèse, Centre National de la Recherche Scientifique UMR 5288, Université de Toulouse, Université Paul Sabatier, Toulouse, France; ^6^CEA, Genoscope, Laboratoire d'Analyses Bioinformatiques pour la Génomique et le Métabolisme, Université d'Evry, Centre National de la Recherche Scientifique-UMR 8030, Evry, France; ^7^CEA, Institut de biologie François-Jacob, Genoscope, Laboratoire de Biologie Moléculaire pour l'Etude des Génomes, Evry, France; ^8^Institute for Integrative Biology of the Cell, CEA, Centre National de la Recherche Scientifique, Univ. Paris-Sud, Université Paris-Saclay, Gif-sur-Yvette, France

**Keywords:** *Vibrio tapetis*, *Venerupis philippinarum*, comparative genomics, pathogenicity, T4SS, pangenome, core genome

## Abstract

The Brown Ring Disease (BRD) caused high mortality rates since 1986 in the Manila clam *Venerupis philippinarum* introduced and cultured in Western Europe from the 1970s. The causative agent of BRD is a Gram-Negative bacterium, *Vibrio tapetis*, which is also pathogenic to fish. Here we report the first assembly of the complete genome of *V. tapetis* CECT4600^T^, together with the genome sequences of 16 additional strains isolated across a broad host and geographic range. Our extensive genome dataset allowed us to describe the pathogen pan- and core genomes and to identify putative virulence factors. The *V. tapetis* core genome consists of 3,352 genes, including multiple potential virulence factors represented by haemolysins, transcriptional regulators, Type I restriction modification system, GGDEF domain proteins, several conjugative plasmids, and a Type IV secretion system. Future research on the coevolutionary arms race between *V. tapetis* virulence factors and host resistance mechanisms will improve our understanding of how pathogenicity develops in this emerging pathogen.

## Introduction

Almost 16 million metric tons of molluscs are cultured each year on a global scale (FishStatJ 2014). In Europe, mollusc aquaculture is a traditional, well-established industry, essential to the socio-economic development of most coastal regions. A number of diseases, however, represent an important threat to this industry (Paillard et al., 2004; Travers et al., 2015). This is the case of the Brown Ring Disease (BRD), which has plagued French aquaculture since 1986, especially the production of *Venerupis philippinarum*, a species native of Japan and introduced in the 1970s (Flassch and Leborgne, 1992).

BRD develops following infection by the bacterium *Vibrio tapetis* and is characterized by the formation of a conchiolin deposit between the edge of the mollusc shell and the pallial line (Paillard et al., 1989; Paillard and Maes, 1994). A number of pathogenic strains have been isolated from diseased clams in France, Spain, the United Kingdom, and Norway (Paillard and Maes, 1990; Paillard et al., 1994, 2008; Novoa et al., 1998; Allam et al., 2000b), as well as from other marine animals, in particular fish such as *Symphodus melops* and *Hippoglossus hippoglossus* in Norway and Scotland (Jensen et al., 2003; Reid et al., 2003). Additional *V. tapetis* strains isolated from the fish *Dicologoglossa cuneata, Solea solea, Genypterus chilensis*, and *Paralichthys adspersus* have been recently proposed as potential causative agents of emergent diseases in Belgium, Spain, England, and Chile (López et al., 2011; Declercq et al., 2015; Levican et al., 2016).

Several *V. tapetis* strains have been studied to characterize the mechanisms underlying the bacterial pathogenicity, including virulence factors, but also the host immune response (Allam et al., 2000a, 2001). Toxins, such as haemolysin and cytotoxins, represent the main virulence factors hitherto identified (Borrego et al., 1996) but other proteins related to virulence have also been identified. For example, null mutants of *djlA*, which encodes an inner membrane chaperone involved in *Legionella* sp. pathogenesis, display a loss of cytotoxic activity against *V. philippinarum* hemocytes *in vitro* (Lakhal et al., 2008). Additionally, VirB4, a component of the Type IV secretion system (Juhas et al., 2008; Christie et al., 2014), is systematically present in the genome of pathogenic *V. tapetis* strains but absent from non-pathogenic strains (ie. not able to reproduce BRD significantly after *in vivo* pallial challenge in *V. philippinarum*) (Bidault et al., 2015).

The complete repertoire of genes associated with *V. tapetis* pathogenicity remains, however, largely unknown, despite the availability of the full sequence of the CECT4600^T^ pVT1 plasmid (Erauso et al., 2011), the identification of molecules involved in pathogen recognition, biomineralization, and cytoskeleton disruption in the transcriptome of the Manila clam (Brulle et al., 2012; Jeffroy et al., 2013; Allam et al., 2014), and the identification of extracellular enzymes, e.g., endochitinases, lipases, and proteases, in the secretome of *V. tapetis* (Madec et al., 2014).

Comparative genomics has become a powerful tool to better understand the pathogenic potential of marine microbes (Medini et al., 2008; Haft, 2015), revealing a fraction of the virulence repertoire in some *Vibrio* genomes (Goudenège et al., 2013; Cordero and Polz, 2014). In the present study, we sequenced and analyzed the first high quality complete genome of a *V. tapetis* pathogenic strain, i.e., CECT4600, and compared it to 16 additional draft genomes of strains isolated from a broad a range of hosts, geographic locations and sampling times (from 1988 to 2008). Two of these strains, namely HH6087 and RP2-3, were recently released as part of a study exploring the long-term persistence of ancient DNA in mollusc shells (Der Sarkissian et al., 2017). Combining comparative genomic analysis and functional information assessing strain virulence using *in vivo* and *in vitro* infection experiments allowed us to further our understanding of the genomic structure and genetic variations underlying *V. tapetis* pathogenicity.

## Materials and methods

### Strains isolation and infection challenge procedures

The *V. tapetis* strains used for genomic analysis are described in Table 1. These were mostly isolated from bivalves showing signs of BRD (*V. philippinarum, Venerupis decussatus, Polititapes aureus, Polititapes rhomboides, Dosinia exoleta*, all belonging to the *Veneridae family*), or from asymptomatic bivalves (*Cerastoderma edule)* but living close to *V. philippinarum* culture areas affected by BRD. All the strains were isolated following the procedures described in Maes and Paillard (1992). Briefly, molluscs were washed with 70% ethanol, dried, and opened aseptically by cutting adductor muscles. The pallial and extrapallial fluids were collected to inoculate Zobell agar plates (4 g/L of peptone, 1 g/L of yeast extract, 0.1 g/L of ferric phosphate, 30 g/L of sea salts, 15 g/L of agar). Additionally, two strains, LP2 and HH6087, were isolated respectively from the posterior kidney of the fish *S. melops* (Jensen et al., 2003) and from the head kidney samples of *H. hippoglossus* (Reid et al., 2003). These strains were kindly provided by Ø. Bergh and H. Birkbeck, respectively.

**Table 1:** General features of *Vibrio tapetis* group.

**Strains**	**Host**	**Country, place, year and people who isolated the strain**	**References**	**Size (Mb)**	**Content GC%**	**CDSs**	**RNAs**
*V. tapetis* CECT4600^T^, CIP 104856 ^T^ B1090^T^ ATCC70075 ^T^	*Venerupis philippinarum*	France, Landeda, (10/1990, Paillard & Maes)	Borrego et al., 1996	3.7/1.8/ 0.8^*^	43.60/43.73/45.46	5,557	97
*V. tapetis* IS-1, VP1	*V. philippinarum*	France, Landeda, (09/1988, Paillard & Maes)	Paillard and Maes, 1990, 1994, 1995	5.5	43.57	5,418	73
*V. tapetis* IS-5	*V. philippinarum*	France, Landeda, (10/1991, Paillard & Maes)	Borrego et al., 1996	5.8	43.54	5,760	73
*V. tapetis* IS-7	*V. philippinarum*	France, Quiberon, (04/1990, Paillard & Maes)	Borrego et al., 1996	5.8	43.53	5,751	74
*V. tapetis* IS-8	*Polititapes aureus*	France, Quiberon, (04/1990, Paillard & Maes)	Borrego et al., 1996	5.9	43.53	5,743	73
*V. tapetis* IS-9	*Cerastoderma edule*	France, Quiberon, (04/1990, Paillard & Maes)	Borrego et al., 1996	5.8	43.53	5,784	50
*V. tapetis* RP2.3	*V. philippinarum*	France, Landeda, (10/1991, Paillard & Maes)	Borrego et al., 1996	5.8	43.61	5,886	71
*V. tapetis* RP8.17	*V. philippinarum*	France, Landeda, (10/1991, Paillard & Maes)	Borrego et al., 1996	5.7	43.57	5,638	75
*V. tapetis* RP9.7	*V. philippinarum*	France, Landeda, (10/1991, Paillard & Maes)	Borrego et al., 1996	5.6	43.56	5,453	74
*V. tapetis* RP11.2	*V. philippinarum*	France, Landeda, (10/1991, Paillard & Maes)	Borrego et al., 1996	5.7	43.57	5,639	74
*V. tapetis* GDE	*Dosinia exoleta*	France, Glenan islands, (03/2003, Le Chevalier & Paillard)	This study	5.7	43.72	5,776	71
*V. tapetis* GTR-I	*Polititapes rhomboides*	France, Glenan islands, (03/2008, Le Chevalier & Paillard)	This study	5.7	43.69	5,538	53
*V. tapetis* P16B	*V. philippinarum*	France, Morbihan Gulf, (09/1995, Paillard & Maes)	Paillard et al., 1997	5.6	43.52	5,581	74
*V. tapetis* UK6	*V. philippinarum*	England, Poole Harbour, (05/1997, Allam & Paillard)	Allam et al., 2000b	5.1	43.53	5,618	67
*V. tapetis* RD0705	*V. decussata*	Spain, Galicia, (12/1992, Maes & Paillard)	Novoa et al., 1998	5.6	43.56	5,644	54
*V. tapetis* subsp. *britannicus* HH6087	*Hippoglossus hippoglossus*	United Kingdon, Scotland, (10/2001, Reid & Birbeck)	Reid et al., 2003	5.6	43.92	6,097	77
*V. tapetis* LP2	*Symphodus melops*	Norway, Bergen, (10/1999, Jensen & Bergh)	Jensen et al., 2003	5.6	43.69	5,643	49

* *Chromosome I/Chromosome II/Plasmid*.

### Virulence assays

*In vivo* pathogenicity tests of *V. tapetis* strains were carried out using two different inoculation procedures in *V. philippinarum*. The first procedure was tailored to test the ability of *V. tapetis* strains to reproduce BRD symptoms. Inoculation was performed following the standardized protocol described by Paillard and Maes (1994). Briefly, a total of 10^7^ bacteria were injected in the pallial cavity of juvenile clams (15–20 mm) that were maintained for 4 weeks, at 14°C, without feeding and water renewal. Each experimental batch consisted of 100 or 50 clams in duplicate. BRD prevalence has been measured 30 days after each *V. tapetis* strains challenge. *In vitro* hemocyte cytotoxicit*y* assays were performed as described by Choquet et al. (2003). This test was performed with all the 17 *V. tapetis* strains at the same time and with the same pool of hemocytes. The non-adherent hemocyte ratio corresponds to the number of non-adherent hemocytes incubated with bacteria divided by the number of hemocytes incubated with filter-sterilized seawater (FSW). A non-adherent cell ratio above 1 represents a cytotoxic effect of the tested bacteria (Choquet et al., 2003). For each of the two pathogenicity tests, *V. splendidus* (ATCC 25914) and FSW were used in the same conditions (negative controls).

### Genome sequencing and assembly

For the 17 *V. tapetis* strains, total DNA was isolated from cultures by lysis of the cells with sodium dodecyl sulfate, EDTA and lysozyme, and extraction was done with a phenol/chloroform solution (Sambrook et al., 1989). The complete genome sequence of *V. tapetis* CECT4600^T^ was obtained using two sequencing technologies: (1) a Sanger library sequencing leading to a 4-fold coverage; (2) a 454-single read library sequencing leading to a 16-fold coverage (in the framework of the Vibrioscope project, Genoscope, Resps. D. Mazel and F. Le Roux). The draft genome of strain LP2, was also performed at Genoscope using 454 GS FLX Titanium paired end sequencing (in the framework of a partnership project of LEMAR). Each of the 15 remaining *V. tapetis* strains was processed in the post-PCR facilities of the Center for GeoGenetics, University of Copenhagen, Denmark. A total of 1 μg of DNA was sheared using a Diagenode Bioruptor ultrasonicator (four cycles of: 15 s at high intensity; 90 s off), and built into a blunt-ended DNA library for Illumina shotgun sequencing as in Orlando et al. (2013). We used the NEBNext Quick DNA Library Prep Master Mix Set (New England BioLabs) with the following conditions: 12°C for 20 min then 37°C for 15 min for end-repair, 20°C for 20 min for ligation (0.5 μM Illumina adapter final concentration), 37°C for 20 min then 80°C for 20 min for fill-in. A volume of 12.5 mL library was amplified by PCR in a 50 μL reaction mix containing: 5 units AmpliTaq Gold, 1X Gold Buffer, 4 mM MgCl_2_ (ThermoFisher), 1 mg/mL BSA, 0.25 mM each dNTP, 0.5 μM Primer inPE1.0 (5′-AAT GAT ACG GCG ACC ACC GAG ATC TAC ACT CTT TCC CTA CAC GAC GCT CTT CCG ATC T-3′), and 0.5 μM Illumina 6 bp-indexed (“I”) primer (5′-CAA GCA GAA GAC GGC ATA CGA GAT III III GTG ACT GGA GTT CAG ACG TGT GCT CTT CCG-3′). Thermo-cycling conditions were: activation at 92°C for 10 min; 7–9 cycles of: denaturation at 92°C for 30 s, annealing at 60°C for 30 s, elongation at 72°C for 30 s; final elongation at 72°C for 7 min. The indexed DNA libraries were pooled and sequenced in single-end mode (94 cycles) on an Illumina HiSeq2500 platform at the Danish National High-Throughput DNA Sequencing Center, with depths of coverage ranging from 50 to 200X. Sequence reads were trimmed by using AdapterRemoval v2.1.5 (Schubert et al., 2016). *De novo* assembly was performed using the SPAdes assembler (v3.7.0, Bankevich et al., 2012) and four k-mer sizes (21, 33, 55, and 77), allowing mismatch correction. QC was performed using QUAST. Downstream analyses were limited to contigs showing a minimum size of 300 bp.

### Genome annotation, comparative genomics, and core genome phylogeny

Expert annotation of the reference strain CECT4600^T^ was done using the MaGe tools of the MicroScope platform (Vallenet et al., 2017). The other 16 draft genomes were then aligned on the complete genome sequence of *V. tapetis* CECT4600^T^ using ABACAS (Assefa et al., 2009) and annotations were collected from MicroScope (Vallenet et al., 2017). Orthologous clusters were computed using GET_HOMOLOGUES (Contreras-Moreira and Vinuesa, 2013), a software relying on three clustering algorithms. We defined the core genome as the consensus of these three algorithms, using a maximal e-value of 1e-05 and a minimum coverage of 75% in pairwise alignments. The pangenome was defined using the sole OrthoMCl clustering algorithm instead of the three implemented in GET_HOMOLOGUES (Contreras-Moreira and Vinuesa, 2013). Circular comparison of the genomes was carried out to identify the genome architecture and the highly conserved and divergent regions using *V. tapetis* CECT4600^T^ as reference and the BRIG software with results plotted as series of lanes, all colored according to BLAST alignment of coding sequences (Alikhan et al., 2011). To perform phylogenetic analyses, we retrieved the single-only copy of core genes following alignment of each individual orthologous gene cluster with Muscle v6.1 (Edgar, 2004). The concatenated alignment was used to construct a core genome maximum-likelihood (ML) phylogenetic tree using the LG amino acid evolutionary model with an estimated gamma distribution with four categories using PhyML (Guindon et al., 2010). Robustness of branching was estimated with 1000 bootstrap pseudoreplicates. The resulting dendrogram was visualized using Figtree v. 1.4.2 (http://tree.bio.ed.ac.uk/).

### Defining pan and core genome size

The size of the core genome and the pangenome were estimated following Tettelin et al. (2005), by fitting an exponential decay function on the distribution of shared genes identified after sequential addition of each new genome sequence. The core genome was fitted by the functions F_c_ = Ω + k_c_exp[−n/τ_s_] and F_s_ = tg(θ) + k_s_exp[−n/ τ_s_], where Ω is the extrapolated core genome size and tg(θ) represents the extrapolated rate of growth of the pangenome size. We adopted the pangenome concept proposed by Koonin and Wolf (2008). In this definition, the distribution of the prokaryotic genes is not limited to two groups only (core and accessory genes), but includes conserved gene core (genomes ≥ 16), the shell of moderately common genes (2 > genomes < 17), and the cloud of genes shared by a small number of organisms (≤2 genomes).

### Identifying type IV secretion system and secondary metabolites

The Type IV secretion system was predicted by CONJscan-T4SSscan (Guglielmini et al., 2011), a tool which scans a set of protein sequences associated and co-localized with VirB4 using HMM profiles. The secondary metabolites biosynthesis gene clusters were identified and annotated using Antismash (Weber et al., 2015).

### Genome accession numbers

The complete genome of *V. tapetis* CECT4600^T^ and the draft genomes of all other *V. tapetis* strains are available from the European Nucleotide Archive (project PRJEB22962).

## Results and discussion

### General features of *V. tapetis* genomes

**Figure 1:**
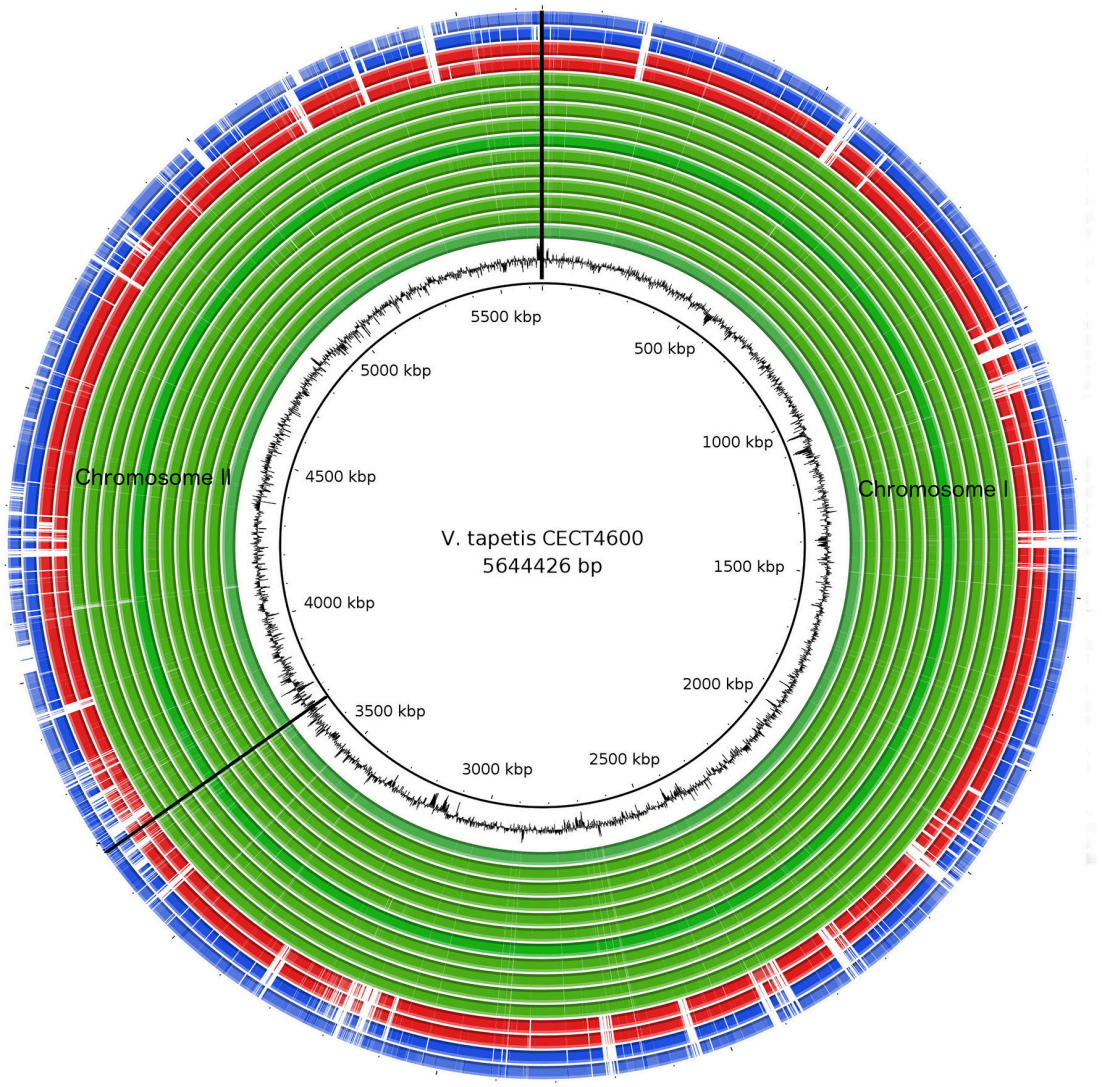
Comparison of *V. tapetis* genomes by blastn analysis. The inner most circle represents the reference genome *V. tapetis* CECT4600^T^. The second internal black circle represents the GC content. The green lanes represent genomes isolated from clams and native cockles before the year 1999 (IS1, IS5, IS7, IS8, IS9, P16B, RD0705, RP2.3, RP8.17, RP11.2, and UK6); red lanes, GDE and GTR-I strains isolated after 2000 from native clams *Dosinia exoleta* and *Polititapes rhomboides*, respectively; blue lanes, strains isolated after 1999 (HH6087, LP2) from fish (*Hypoglossus hypoglossus, Symphodus melops*). Genomic regions corresponding to the absence of genes in a given strain are in white.

In this study, we generated the complete *de novo* genome assembly of the *V. tapetis* strain CECT4600^T^, and delivered *de novo* genome draft assemblies for 16 additional *V. tapetis* strains (genomic data from two of them were previously released in Der Sarkissian et al., 2017), this represents a total of 12 strains isolated from molluscs belonging to the *Venerupis* genus, three from other molluscs of the *Veneridae* family (*Cerastoderma edule, P. rhomboides, D. exoleta*), and two from fish (*H. hippoglossus* and *S. melops*). The complete genome of *V. tapetis* CECT4600^T^ used as the reference consists of two circular chromosomes of 3.7 Mb (chromosome I, %GC = 43.60) and 1.8 Mb (chromosome II, %GC = 43.73), for which a total number of 3,664 and 1,833 open reading frames (ORFs) were predicted, respectively. Additionally, the CECT4600^T^ strain carries the 88 Kb (%GC = 45%) pVT1 plasmid, which presents 88 ORFs (Erauso et al., 2011). All the other 16 *V. tapetis* strains harbors the pVT1 plasmid. Similar genome sizes (5.5–5.9 Mb) and numbers of predicted genes (5,418–6,097 protein coding DNA sequences, CDS) were observed in the draft genomes of all other strains (Table 1). On average, the genomes of *V. tapetis* encode 5,678 proteins, and show moderate genetic conservation of ~68%. Orthologous gene clustering revealed that CECT4600^T^ and the strains isolated from clams and cockles (*Venerupis, Cerastoderma*, and *Polititapes* genus) share more genes [on average 4,768 out of 5,678 (~83%)] than strains isolated from fish [on average 4,142 out of 5,678 (~72%)]. To obtain a global comparison of the genomes, we constructed a circular blast atlas. *V. tapetis* genomes were compared by blastn alignment using *V. tapetis* CECT4600^T^ as the reference. The blast atlas showed the highest conservation of genes between species isolated from clams before year 2000, in comparison to strains isolated from clams after year 2000 and those isolated from fish (Figure 1, red and blue lanes). Interestingly, genome alignments for these strains showed multiple gaps, illustrating that genetic variations are not evenly distributed along the genome, underscoring the importance of genomic rearrangements such as deletion and horizontal gene transfers in evolution (Figure 1).

### Phylogenetic analysis based on the core genome

**Figure 2:**
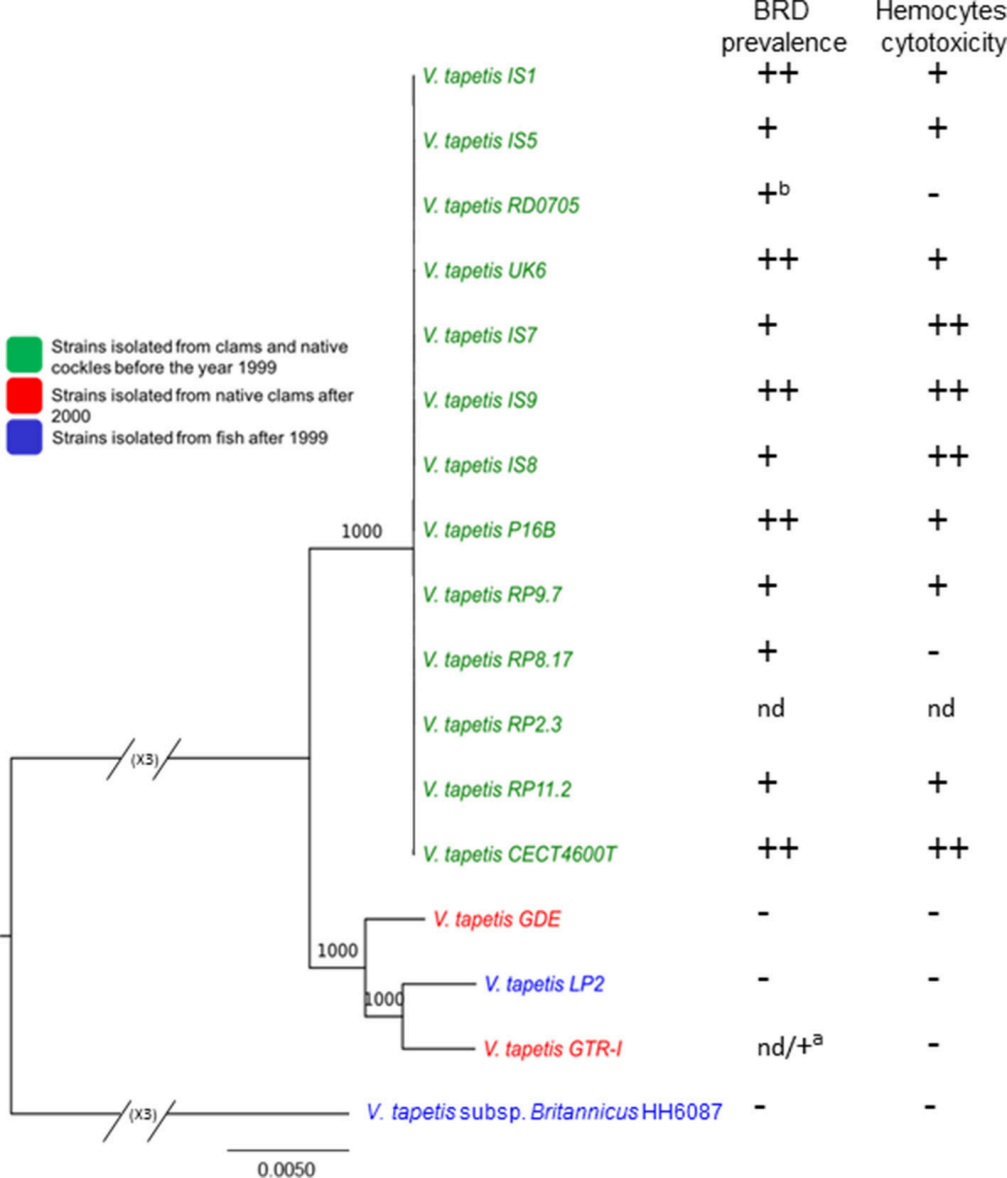
Maximum-likelihood tree of *V. tapetis* genomes based on single-copy genes of the *V. tapetis* core genome and pathogenicity assays. *V. tapetis* strains can be divided in three groups. The first group includes the virulent strains (green), while the two other groups comprise the less virulent strains (red and blue). Note that the branching pattern for the first group cannot be visible on this figure, due to the huge difference with the HH6087 strain; a specific tree for these strains is given as additional data (Figure S1). The numbers at the nodes indicate the levels of bootstrap support based on 1,000 replicates. Hosts and years of isolates are indicated in the legend. On the right of the figure, BRD prevalence in clams (*in vivo*) after pallial inoculation (average obtained from 1988 to 2007) and hemocyte cytotoxic assay (*in vitro*) performed in 2014 are given. nd: not determined, ^a^Pathogenicity of GTR-I strain after pallial cavity inoculation in *Polititapes rhomboides*. ^b^Obtained from both this study and Novoa et al. (1998). BRD prevalence; (−): average lesser than 20%, (+): 20%<average< 65%, (++): average greater or equal to 65%. Hemocyte cytotoxic assay; (−): ratio ≤1, (+): 1<ratio<1,5, (++): ratio>1,5.

We performed a phylogenetic analysis based on the 2,577 single-copy genes present in the *V. tapetis* core genome (see the pan-/core-genome paragraph below). This showed that the reference strain CECT4600^T^ clustered together with 12 additional strains, namely IS1, IS5, IS7, IS8, IS9, UK6, RD0705, RP2.3, RP8.17, RP9.7, RP11.2, and P16B. This cluster showed limited genetic variation (Figure 2; Figure S1), suggesting a recent common origin for those pathogenic strains that infected clams and cockles prior to 2000 in France, the UK, and in Spain. This is consistent with the phylogenetic tree based on MLSA (Figure S2). A second cluster included 3 more divergent strains (GDE, GTR-I, and LP2), isolated in 1999 and 2000 from a range of hosts. Finally, the HH6087 strain appeared divergent from all other strains. The taxonomic position of this strain has been debated since it was first isolated in 2001 (Reid et al., 2003). In spite of an early assignment as a *V. tapetis* member based on almost perfect 16S rRNA sequence identity with the CECT4600^T^ strain and additional common biochemical features (Reid et al., 2003), another study on the basis of a 9-gene MLSA showed that both strains were more divergent (Balboa and Romalde, 2013), consistent with our results. DNA-DNA hybridization experiments revealed reassociation values of 69.5–74.8% (Balboa and Romalde, 2013), thus close to the 70% threshold commonly used for species determination (Wayne et al., 1987; Tindall et al., 2010), which supported its description as a new subspecies named *V. tapetis* subsp. *britannicus* (Balboa and Romalde, 2013). Additional members of the HH6087 cluster are needed before the taxonomic status of this group as a subspecies or as a new species of its own can be determined.

This phylogenetic analyses showing closer ancestry between CECT4600^T^ and clam/cockle strains than fish strains is in agreement with previous results on global genomic comparison of *V. tapetis* genomes (Figure 1).

### Virulence assays

In order to test the potential virulence of the strains, we used the BRD-induction challenge that appears as the most discriminant *in vivo* pathogenicity test between the *V. tapetis* strains. It mimics the natural transmission of bacteria into pallial fluids through the siphons, the adhesion and proliferation of virulent *V. tapetis* within the shell, and induces secretions linked to BRD development (Paillard, 2016). Using this *in vivo* BRD induction challenge, all strains isolated between 1988 and 1998 were able to induce BRD development in *V. philippinarum*, with prevalence rates ranging from 39 to 84% (Figure 2, Figure S3). Strains CECT4600^T^, IS1, IS9, UK6, and P16B showed the highest BRD prevalence rates (above 70%). The strains isolated from another clam species, *D. exoleta* (GDE), and from fish (HH6087 and LP2) showed limited prevalence rates (about 15%). It is noteworthy that approximately ~11,500 clams were inoculated for all these assays and the mortality rate was always limited to 3%. Using the *in vitro* cytotoxic bioassays based on the reduction of adhesion properties of *Ruditapes philippinarum* hemocytes after *V. tapetis* contact, the fours trains LP2, HH6087, GDE, and GTR-I showed also low toxicity compared to the other *V. tapetis* strains (Figure 2). These results are consistent with those already obtained for some strains of *V. tapetis* and in particular for LP2 (Choquet et al., 2003). In conclusion, virulence assays allowed to show that the four *V. tapetis* strains LP2, HH6087, GDE, and GTR-I display a weak virulence against the manila clams.

### *Vibrio tapetis* pangenome and core genome size

**Figure 3:**
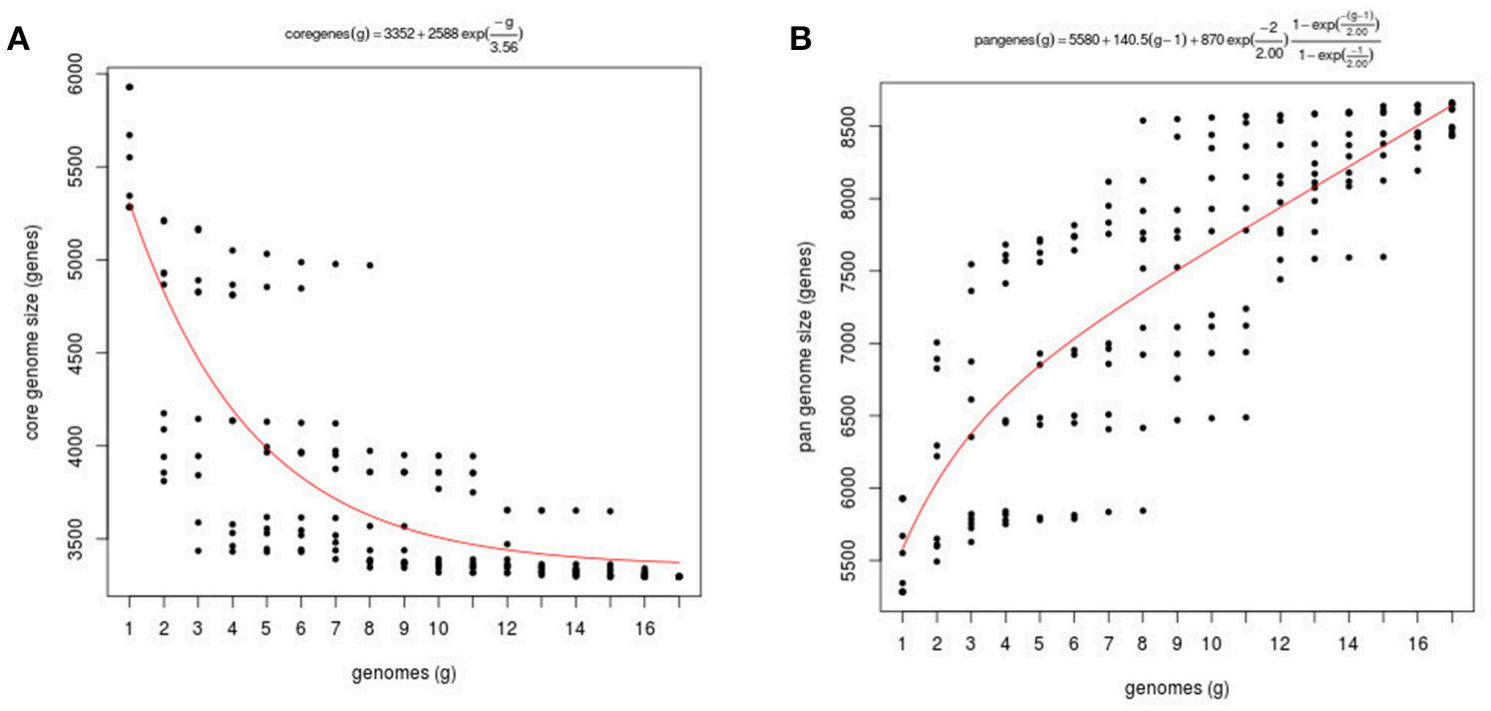
Core and pangenome of *V. tapetis*. **(A)** Plot of the exponential decay model of Tettelin et al. (2008) fitted to the core genome data using the OMCL algorithm. **(B)** Estimation of pangenome size of 17 taxa through function proposed by Tettelin et al. (2005).

We estimated the *V. tapetis* pangenome, that comprises both the core genome, i.e., the set of genes found across all strains, the variable (flexible) genome consisting of genes absent in one or more strains, and genes unique to some strains. The pangenome and core genome sizes were calculated using the fitting regression model of by Tettelin et al. (2008). As a rule, the number of genes in the core genome depends of the number of samples included in the analysis and the organisms. For example, the core genome of *Vibrio vulnificus*, calculated from the analysis of 17 genomes, contains 3,068 genes, a number similar to the one we found in *V. tapetis* (Koton et al., 2015). By contrast, a study based on 42 *V. cholerae* genomes identified a core genome containing 2,089 genes (Orata et al., 2015). As expected, our core genome size tended to decrease when more genomes were considered in the analysis, with a concomitant increasing importance of the variable genome, the core final size approaching 3,352 genes (Figure 3A). According to the Tettelin model, for a given species, the pangenome can be classified as “*open*” or “*closed*,” whether or not its size increases with the addition of genomes to the analysis (Medini et al., 2005; Tettelin et al., 2008). For instance, previous studies showed that the *Streptococcus agalactiae* pangenome increases on average by 33 genes per genome added to the analysis, and that of *Pseudomonas aeruginosa* increases by 29 (Tettelin et al., 2008; Mosquera-Rendón et al., 2016). We found that the *V. tapetis* pangenome increased by an average of 140 genes following the inclusion of one single genome in the analysis (Figure 3B). This indicates that the *V. tapetis* pangenome is more open than that characterized for important human pathogens, probably pertaining to differences in the spectrum of their respective habitats and environmental exposures. Future studies providing additional *V. tapetis* genomes are likely to lead to the discovery of additional pangenome genes.

### Functional classification of the core, cloud, and shell genomes

**Figure 4:**
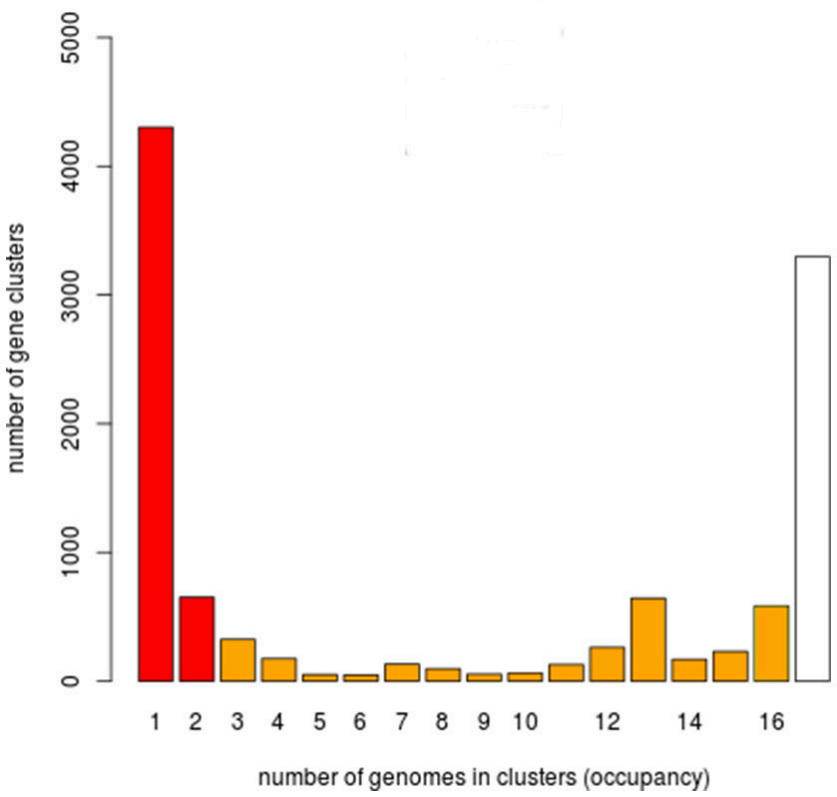
Histogram for the prevalence of genes of the *V. tapetis* pangenome. 11,213 genes of the *V. tapetis* pangenome were distributed according to their frequencies across all the strains analyzed. White, orange, and red bars represent the core, shell and cloud genomes, respectively.

We next carried out a functional analysis of the core, shell, and cloud genomes in order to identify the diversity and composition of the global gene repertoire of *V. tapetis* strains (see Material and Methods). A total number of 11,213 genes were identified in the *V. tapetis* pangenome. The core genome contained 3,881 genes, while shell and cloud genomes contained 2,378 and 4,954 genes, respectively (Figure 4). In total, 82% of the genes included in the core genome, as well as 47% of the shell and 30% of the cloud genomes, could be assigned to a COG category. Some COG categories were over-represented in the core genome, such as “Amino acid transport and metabolism” (E), “Coenzyme transport and metabolism” (H), “Energy production and conversion” (C), and “Inorganic ion transport and metabolism” (P). The categories that were over-represented in the shell genome were “Cell Motility” (N), “Intracellular trafficking, secretion and vesicular transport” (U), “Lipid transport and metabolism” (I) and “Transcription” (K), while those pertaining to the cloud genome were “Cell wall/membrane/envelope biogenesis” (M), “Replication/recombination and repair” (L), and “Defense mechanisms” (V). Exploring the genes of each category, we found several genes of the core genome involved in sulfate and phosphate metabolism. We speculate that these could be involved in scavenging sulfate and phosphate ions from the host, thus providing sulfur, phosphate and/or carbon sources, which could facilitate survival within the host. The shell genome included several genes involved in the conjugative transfer of F-plasmid homologs (*traALEKBV*), several proteins containing GGDEF/EAL domains, proteins involved in the utilization of sialic acid, O-antigen genes, toxin-antitoxin system, and genes involved in biofilm formation (*tad* locus). Finally, the cloud genome consists mainly of genes related to integrative and conjugative elements, prophages and phage-like elements, transposons, insertion sequences, genes encoding acriflavin resistance and genes involved in Type I restriction-modification system (Tables S2, S3).

### Accessing the differences between the *V. tapetis* genomes

Our experimental assays showed that the *V. tapetis* strains belonging to the first monophyletic cluster (Figure 1) are able to induce the development of BRD with a 75% prevalence on average. By contrast, the average prevalence of BRD induction for GDE, LP2, and HH6087 was more limited (<20%, Figure S3). This supports the hypothesis of two independent origins for the strains, either time of isolation (before and after year 2000), or host species specificity. In order to identify the set of genes common to the most virulent strains, we compared the genomes of the 13 virulent strains (CECT4600^T^, IS1/5/7/8/9, RP2.2, RP8.17, RP9.7, RP11.2, UK6, RD0705, and P16B) to those of the 4 strains showing lower virulence (GDE, GTR-I, LP2, and HH6087). A total of 444 protein coding genes (24% of which are annotated as hypothetical) were present in the virulent strains and absent in less virulent strains. These included CTX phage elements, Type IV secretion system (T4SS) components (see below), signal transduction proteins such as GGDEF/EAL family proteins, and proteins encoded by regions similar to the pVT1 plasmid. Importantly, *V. tapetis* strains showing maximal BRD induction levels (Figure S3) display unique putative virulence genes (Table 2), such as genes encoding a Type IV secretion system, genes for toxins (RTX and esterase LpqC), and genes involved in polysaccharide metabolism. The potential contribution of these genes to virulence is discussed in more details below.

**Table 2:** Gene products putatively involved in virulence in *V. tapetis*.

	**Strains**			
**Products**	**CECT4600,IS1/5/7/8, RP2.3, RP8.17,RP9.7,RP11.2 P16B,UK6,RD0705**	**GTR-I**	**GDE**	**HH6087,LP2**
**BIOFILM FORMATION**				
Syp cluster encoded proteins	X	X	X	X
Tad locus encoded proteints	X	X	X	X
Type IV pilin PilA	X	–	X	–
**TOXICITY**				
Accessory cholera enterotoxin	X	–	–	–
Zonular occludens toxin family protein	X	X	–	–
RstA-phage-realted replication protein	X	–	–	–
RstB phage related integrase	X	–	–	–
Von Willebrand factor type A domain	XX	X	X	X
Probable RTX	X	–	–	–
Esterase LpqC	X	–	–	–
**POLYSACCHARIDE**				
Acylneuraminate cytidylyltransferase (NeuA)	X	–	–	–
Oxidoreductase (NeuB)	X	–	–	–
D-glycero-D-manno-heptose 1-phosphate guanosyltransferase (NeuC)	X	–	–	–
Sialic acid biosynthesis protein (NeuD)	X	–	–	–
**PROTEASE**				
Subtilisin-like serine protease	X	–	X	–
Outer membrane stress sensor protease DegQ, serine protease	X	X	X	X
**SECRETION**				
HlyD family secretion protein	XX	X	–	X
Colicin V secretion ATP-binding protein CvaB	X	X	X	X
Type VI secretion system	X	X	X	X
Type IV secretion system	X	–	–	–

*X-indicates the presence of protein; XX, presence of two copies; –, absence of gene*.

### Host immunity resistance genes

Genes related to catabolism and transport of sialic acid were found in several pathogenic bacteria including *V. cholerae* and *V. vulnificus* (Almagro-Moreno et al., 2009; Lubin et al., 2012; Kim et al., 2013). These were shown to contribute to provide resistance to components of the host innate immune response and the ability to use such components as a nutrient source (Severi et al., 2007). For all of the 17 *V. tapetis* strains included in our study, except HH6087 and LP2 strains, we identified putative orthologs to genes involved in the catabolism and transport of sialic acid, in particular *nanA, nanK, nanE*, and TRAP transporters. We could also identify homologs of the *nanH* gene, which encodes the sialidase enzyme involved in the release of free sialic acid. At the sequence level, *V. tapetis* strains, *V. cholerae*, and *V. vulnificus* show >80% identity in their sialidase genes. This suggests that most of the analyzed *V. tapetis* strains have the capacity to degrade and/or capture sialic acids from their hosts in order to escape recognition by the host immune system. This is in agreement with previous studies showing that the transcripts of sialic acid binding lectins are up-regulated during BRD in clams (Allam et al., 2014).

### Secondary metabolites

All *V. tapetis* strains shared six secondary metabolites biosynthetic gene clusters, except the HH6087 strain, which shows only three clusters. The compounds identified through the antiSMASH prediction tool showed that these clusters should be involved in the synthesis of siderophores, polyketide synthesis (PKS), non-ribosomal peptides synthesis (NRPS), the osmoprotectant ectoine, and polyunsaturated fatty acids (PUFA). The three clusters present in the HH6087 genome contained only clusters related to PUFA, bacteriocin, and siderophore biosynthesis. Finally, one additional cluster related to aryl polyene biosynthesis could be detected only in the IS5, IS7, IS8, and IS9 strains.

#### Siderophores

The NRPS and NRPS-independent clusters that are responsible for the biosynthesis of vanchrobactin and achromobactin-like siderophores were found in all *V. tapetis* strains. By contrast, the HH6087 genome did not contain any gene related to vanchrobactin siderophore biosynthesis. The non-ribosomal biosynthetic pathway of vanchrobactin was previously identified in *V. anguillarum* and *V. campbellii* species (Balado et al., 2006; Dias et al., 2012). The putative NRPS-independent gene cluster showed similarity (>70%) to the achromobactin biosynthesis cluster of *Pseudomonas syringae* pv*. Syringae* (i.e., dimethyl menaquinone methyltransferase, AcsA, AcsB, AcsC, and AscE proteins, Table S1), a pathogen of both monocot and dicot plants (Berti and Thomas, 2009; Ravindran et al., 2015). In *P. syringae* pv*. Syringae* mutants defective for the synthesis of achromobactin siderophores exhibit reduced growth in low-iron conditions (Berti and Thomas, 2009). The exact role of siderophores is still unclear in bacteria-bivalve interactions. However, iron is known as one essential minor element in the bivalve shell (Swinehart and Smith, 1979; Zhang et al., 2003). Presumably the ability of most *V. tapetis* strains to produce and secrete siderophores could induce a competition for iron between bacteria and their hosts. It follows that the presence of siderophores in *V. tapetis* may confer an advantage in host colonization, which remains to be tested through functional experimental assays.

#### Polyketide synthases (PKS)

The presence of PKS genes in both marine animals and microbiota presents a high potential for the production of polyketide secondary metabolites in marine communities/organisms. These metabolites are of paramount importance for human health and the industry, and they have important ecological roles as antibiotics and toxins, and providing chemical defenses against pathogens (Ziemert et al., 2014; Amoutzias et al., 2016). We found a Type-I PKS cluster in all the genomes analyzed, except for the HH6087 strain. This cluster has an average size of 65 Kb, and includes one copy of polyketide synthase (PKs), a non-ribosomal peptide synthetase, transporters, transcriptional regulators, the multidrug toxin extrusion (MATE) family efflux pump YdhE/NorM and a number of thioesterases. We also identified tRNAs at both ends of the cluster, suggesting the possible cluster acquisition through horizontal gene transfer.

#### Arylpolyene

We found an aryl polyene (APEs) cluster in IS5, IS7, IS8, IS9, RP2.3, RP8.17, RP11.2, and RD0705 strains. The aryl polyene products are responsible for the pigmentation of Gram-negative bacteria where they are widely distributed, including in commensals or pathogens of eukaryotic hosts, and could be involved in the protection against exogenous oxidative stress (Cimermancic et al., 2014). APEs are related to the antioxidative carotenoid group (Schöner et al., 2016). The *V. tapetis* APE cluster displays 80% similarity to most of the proteins from the *V. fischeri* APE cluster which confers a yellow pigmentation to wild-type *V. fischeri* ES114 (Cimermancic et al., 2014). Sequence analysis revealed that the APE cluster is likely to have been introduced into the *V. fischeri* ES114 genome as a result of a recent horizontal gene transfer. The presence of this cluster in *V. tapetis* may protect the bacterium from the oxidative stress induced by immune cells during the colonization or infection.

#### Ectoine biosynthesis

We found genes related to the ectoine biosynthesis cluster in all strains, except in the HH6087 genome. Ectoine, a cyclic tetrahydropyrimidine (1,4,5,6-tetrahydro-2-methyl-4-pyrimidinecarboxylic acid) can be considered as a marker for halotolerant bacteria. Intracellular osmolytes accumulation in hyperosmotic environments is a growth support and survival strategy in most living organisms, and acts by controlling the osmotic equilibrium in the cells; compatible solutes can be either neosynthesized or imported by various bacteria (Pichereau et al., 2000; Roberts, 2005). Ectoine accumulation confers a competitive advantage in the growth and survival of *V. parahaemolyticus* and *V. cholerae* in high osmolarity environments (Pflughoeft et al., 2003; Ongagna-Yhombi and Boyd, 2013), therefore the ability of *V. tapetis* strains to accumulate ectoine may pertain to an ecological advantage both in the marine environment and during host colonization.

### Characterization of the lipopolysaccharide O-antigen of *V. tapetis*

**Figure 5:**
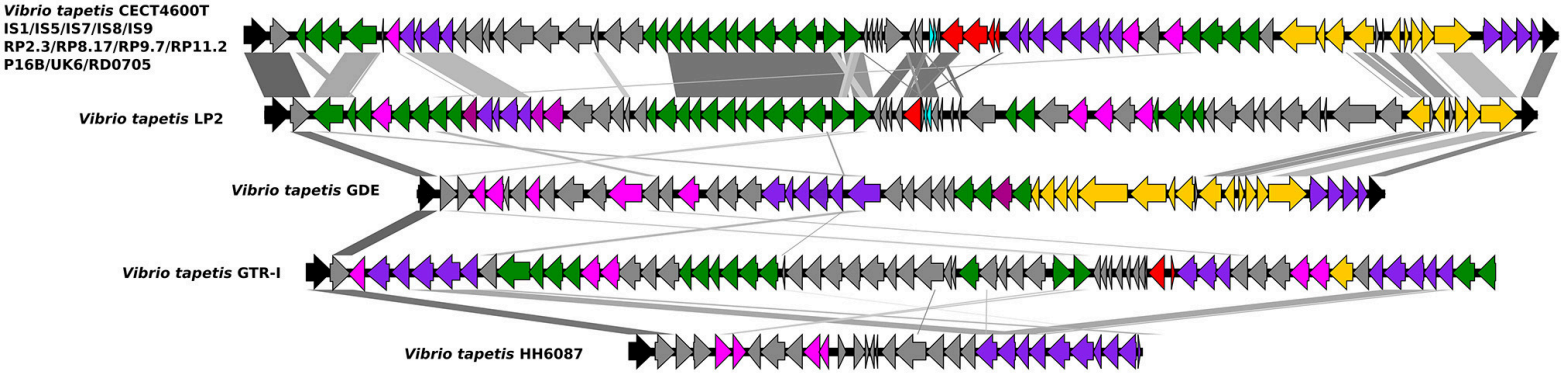
Genetic organization of the O-antigen locus in *V. tapetis* strains. The region is flanked by the *gmhD* and *gpm* genes (black arrows). Positions and orientations of CDSs are indicated with different color, gray arrows: hypothetical proteins and other; acqua arrows: toxin/antitoxin system; red arrows: mobile element proteins; green arrows: genes involved to glycosylation protein and legionaminic acid biosynthesis; yellow arrows: genes involved to capsular polysaccharides biosynthesis and assembly; purple arrows: genes involved to dTDP-rhamnose synthesis and mannose metabolism; fuschia arrows: glycosyltransferases.

The O-antigen biosynthesis gene cluster was identified in all strains investigated and ranged in size from 38 Kb (GDE) to 76 Kb (CECT4600^T^). All *V. tapetis* strains isolated from the *Venerupis* genus displayed the same genetic organization as those isolated from *P. aureus* (IS8) and *C. edule* (IS9), with two main regions separated by a 7.6 Kb region containing ~15 genes encoding hypothetical proteins, a number of mobile elements, and one toxin-antitoxin system. Strikingly, the O-antigen cluster of the remaining *V. tapetis* strains, namely those forming the second phylogenetic lineage (GDE, GTR-I, and LP2), as well as the more divergent strain HH6087, displayed a different organization (Figure 5). The first and second regions of the O-antigen biosynthesis gene cluster are flanked by the *gpm* and *gmhd* genes, and include respectively 32 and 34 genes involved in the production of nucleotide sugars used as building blocks in polysaccharide biosynthesis, glucose, rhamnose, and the unusual legionaminic acid synthesis pathways. The legionaminic acid is a component of lipopolysaccharide in several bacteria and is best known for its presence in the O-antigen of the causative agent of Legionnaires' disease, *Legionella pneumophila* (Glaze et al., 2008). The legionaminic acid belongs to the NulOs family that includes other acids, such as neuraminic and pseudaminic acids. Biochemical and computational analyses revealed that the production of NulOs is functional in several *Vibrio* isolates. *V. vulnificus* clinical isolates expressed higher levels of NulOs than environmental isolates. It was found that role of NulOS in motility, biofilm formation, and pathogenicity to aquatic or terrestrial animals (Lewis et al., 2011). The exact role of the *V. tapetis* O-antigen is still unknown, but *V. fischeri waal* deficient mutants show motility defect, resulting in colonization delays (Post et al., 2012). The O-antigen and the core components of lipopolysaccharides are in direct physical interaction with the surrounding substrates and are thus subject to environmental selective pressures. The structural and gene content variation observed within the *V. tapetis* O-antigen cluster suggests a coevolutionary diversification process in relation to interactions with different hosts.

### Type IV secretion system (T4SS)

**Figure 6:**
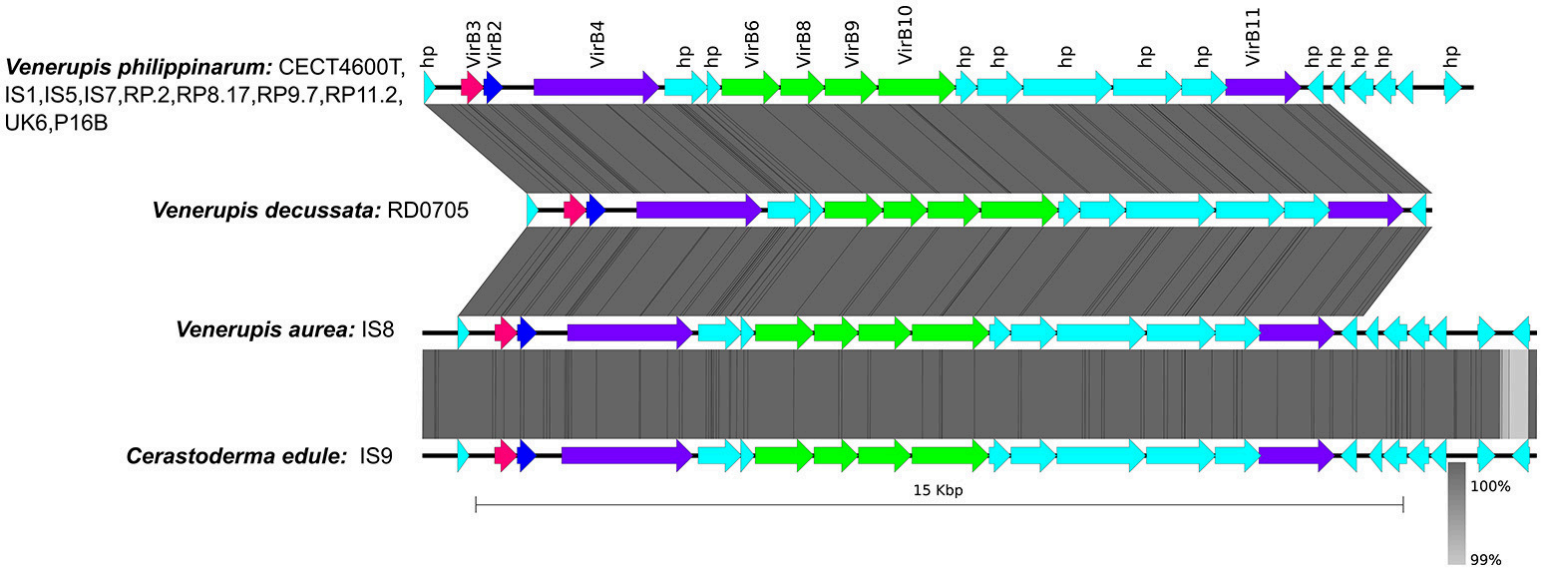
Organization of the Type IV secretion system locus of *V. tapetis* strains isolated from different bivalves. The arrow indicates each CDS of the cluster according to function. Cyan arrows: hypothetical proteins; purple arrows: virB4 and virB11; dark pink: virB3, blue: virB2; green: virB6, virB8, virB9, and virB10.

We found a conserved gene cluster that potentially encodes a Type IV secretion system in 13 of the 17 genomes (Table S4) and was absent in the non-virulent strains GDE, GTRI, LP2, and HH6087 (Figure 6). This finding is in line with recent real time PCR experiments targeting T4SS elements in these strains (Bidault et al., 2015). The T4SS cluster of *V. tapetis* strains consists of the *vir* operon (*virB2, virB3, virB4, virB6, virB8, virb9, virB10, virB11*) plus genes of unknown functions. VirB1 is a lytic transglycosylase that degrades the peptidoglycan cell wall at the site of T4SS assembly. VirB2 and VirB5 are pili components, VirB3 and VirB7 pili associated proteins, VirB4 and VirB11 are nucleoside triphosphatases that provide energy for transfer and VirB6, VirB7, VirB8, VirB9, and VirB10 are the protein components of the transfer channel (Juhas et al., 2008). This system is closely related to the well-characterized phytopathogen *A. tumefaciens* VirB system which mediates the transfer of oncogenic T-DNA into plant cells, inducing tumorous growth of infected plant tissues (Escobar and Dandekar, 2003). However, T4SS can transport a wide variety of compounds from DNA during conjugation to effector proteins, in case of pathogenic bacteria. T4SS have been shown to be essential for virulence in several pathogenic species such as *H. pylori, L. pneumophila*, or *Bordetella pertussis* (Voth et al., 2012). The presence of T4SS gene cluster in *V. tapetis* strains that present a high degree of virulence, and the absence of these genes in strains less virulent, suggest that T4SS have a crucial role in host adaptation and virulence through the delivery of effector proteins in the bivalves.

## Conclusions

Brown Ring Disease has decimated clam cultures in several European countries since the late 1980s. Previous studies focused on the ecological, physiological, and epidemiological characterization of the disease. This paper provides the first study of the genome organization and sequence of the BRD etiological agent, *V. tapetis*. We sequenced and analyzed 17 genomes from *V. tapetis* strains isolated from a broad range of hosts and geographical origins and at different times. We found that the pan-genome of *V. tapetis* consists of 11,253 genes, and that the core, shell, and cloud genomes contain a diversity of putative virulence factor and secondary metabolite pathway encoding genes. Importantly, *V. tapetis* strains showing maximal BRD induction levels display unique putative virulence genes such as a Type IV secretion system gene cluster, genes for toxins (RTX) and proteases. Phylogenetic analysis has shown that the strains isolated from clams before year 2000 cluster together within a monophyletic group despite deriving from a wide range of locations. This analysis also identified two additional groups, encompassing strains isolated from clams after the year 2000 and from fish, respectively, with the strain HH6087 being the most divergent, both at the sequence and structural levels. These two groups displayed limited BRD prevalence rates. In addition, a T4SS gene cluster has been identified only in the genomes of *V. tapetis* strains virulent to *V. philippinarum*, strongly suggesting a role for this T4SS system in pathogenicity. To the best of our knowledge, this study is the first to describe a complete T4SS gene cluster in a vibrio pathogenic to mollusc.

## Author contributions

CP conceived and coordinated the study. AB performed molecular analyses. CM, VB, and SM performed genome sequencing and assembly of *V. tapetis* CECT4600 and LP2 strains at Genoscope (Evry, France). AJ performed the expert annotation of *V. tapetis* CECT4600 reference strain on Mage. CDS and LO performed molecular experiments related to the sequencing of 15 *V. tapetis* genomes at the Center for GeoGenetics (Copenhagen, Denmark). VP performed the genome assembly of 15 *V. tapetis* strains. GD performed computational analyses with input from VP, LO, FT, CT, and CP. GC, PLC, and CP performed virulence assays. GD, VP, and CP wrote the article, with great inputs from LO and AJ.

### Conflict of interest statement

The authors declare that the research was conducted in the absence of any commercial or financial relationships that could be construed as a potential conflict of interest.
