# Supplementary material for: Vibrio tapetis Displays an Original Type IV Secretion System in Strains Pathogenic for Bivalve Molluscs
Source: Front Microbiol. 2018 Feb 19;9:227. doi: 10.3389/fmicb.2018.00227 (PMC5825899; doi:10.3389/fmicb.2018.00227)
Supplement: Supplementary file 1 [file DataSheet1.PDF]

Supplementary Material

Vibrio tapetis displays an original type IV secretion system in strains pathogenic for bivalve molluscs

Graciela Maria Dias[1,2], Adeline Bidault[1], Patrick Le Chevalier[3], Gwenaëlle Choquet[1], Clio Der Sarkissian[4], Ludovic Orlando[4,5], Claudine Medigue[6], Valerie Barbe[6], Sophie Mangenot[7], Cristiane C. Thompson[2], Fabiano L. Thompson[2], Annick Jacq[8], Vianney Pichereau[1], Christine Paillard[1*]

*** Correspondence:** Christine Paillard

1 Supplementary Figures and Tables

1.1 Supplementary Figures

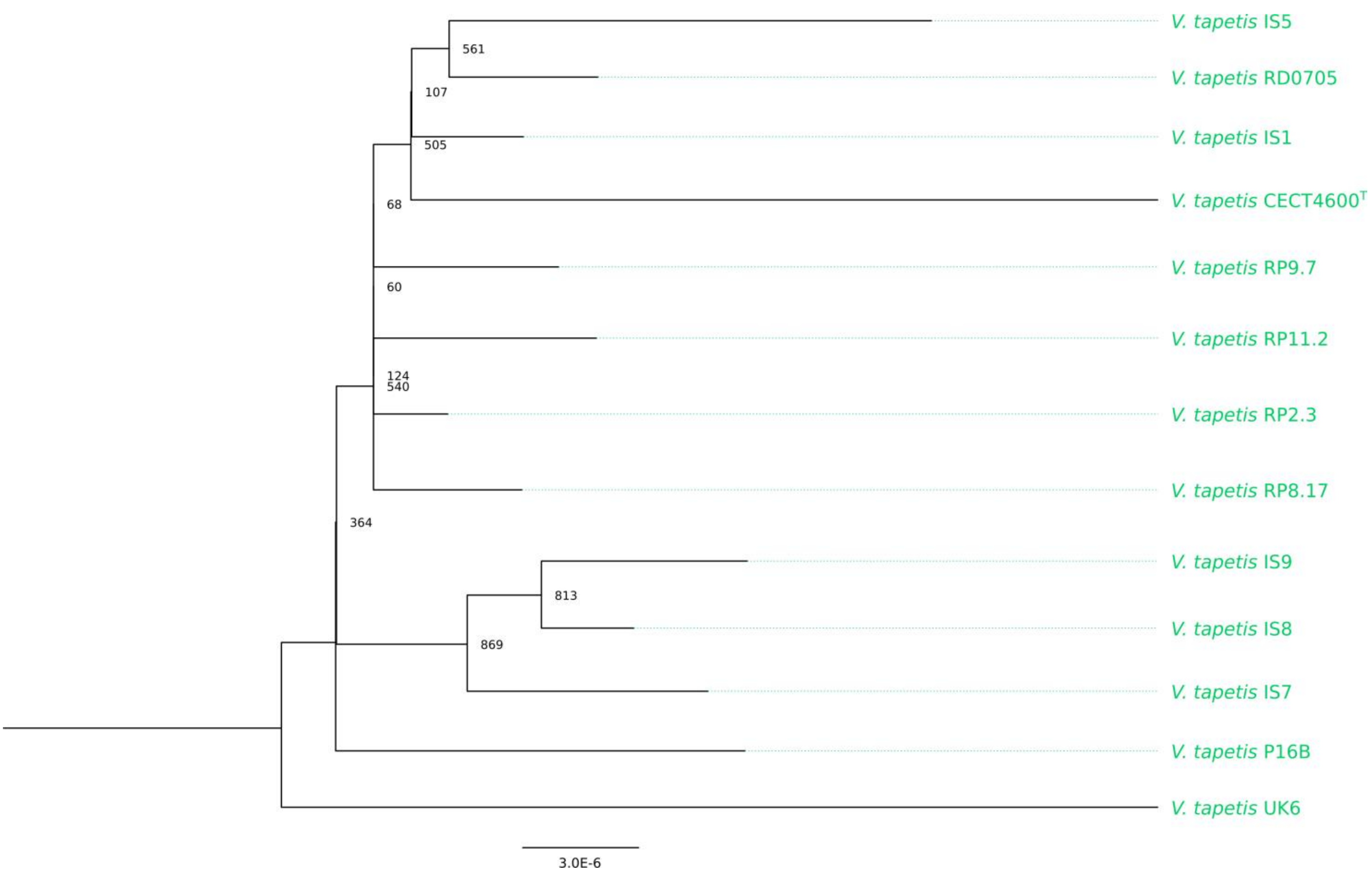

Figure S1. Maximum-likelihood tree of the *V. tapetis* virulent strains, based on single-copy genes of the *V. tapetis* core genome.

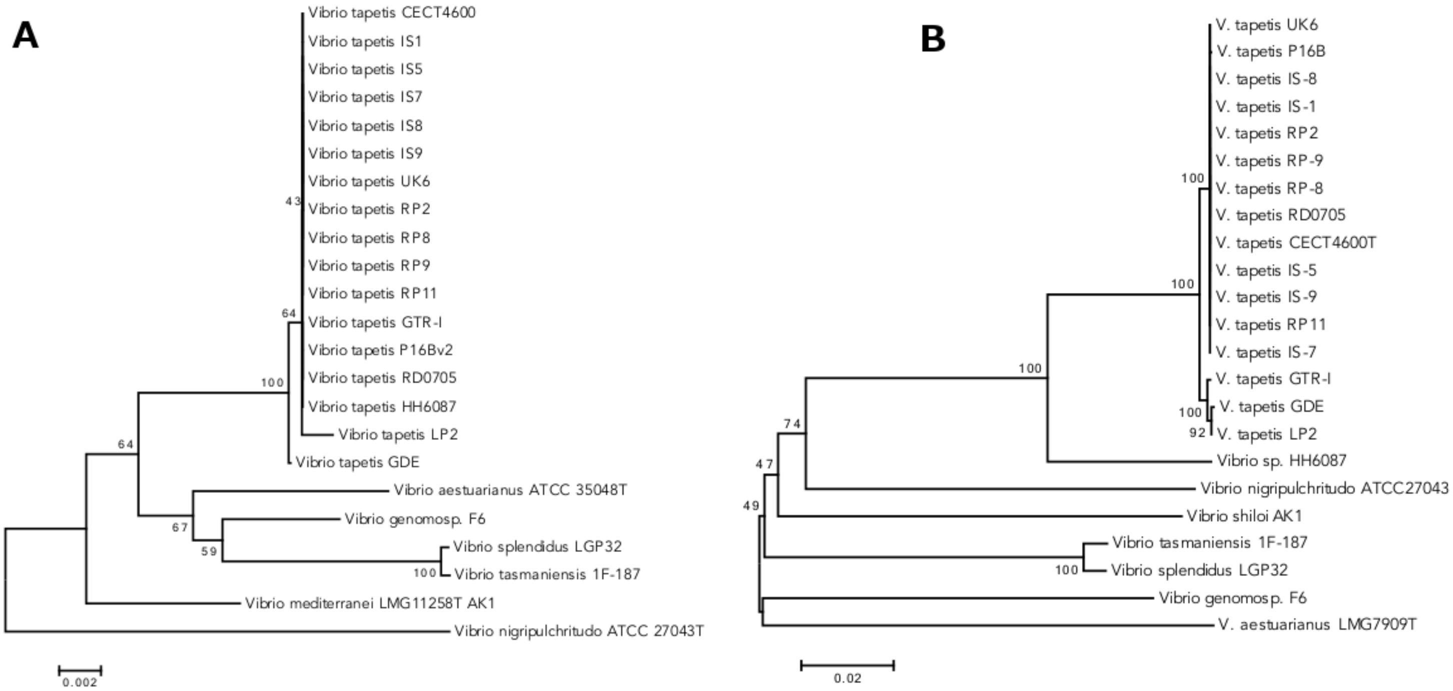

Figure S2. Phylogenetic tree of *V. tapetis* based on 16S rRNA and concatenated *ftsZ, gyrB, mreB, pyrH, rpoA,mreB, rpoA, topA* gene sequences

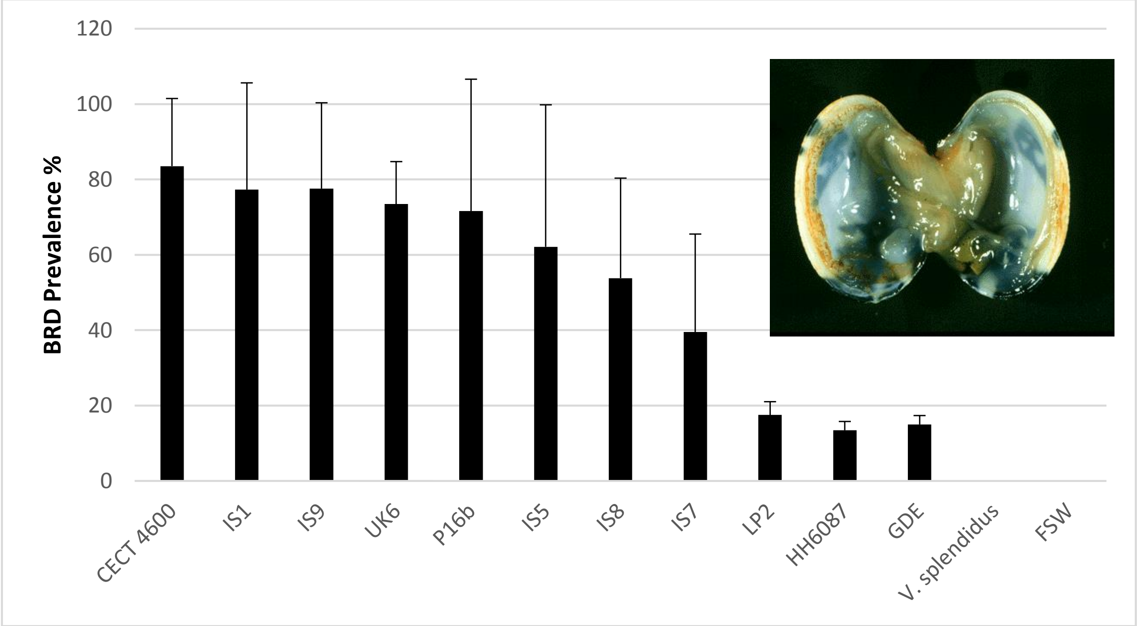

Figure S3. BRD prevalence, 30 days after pallial inoculation of *V. tapetis* strains in *V. philippinarum*. *V. splendidus* ((ATCC 25914) and Sterile Sea Water (SSW) were used as control ones. The mean value was calculated for experiments performed between 1988 to 2007. Error bars represent standard deviation of the mean. Picture of the characteristic symptoms of the BRD, a brown ring

on the peripheric inner face of the valves of *V. philippinarum*, obtained 30 days after experimental challenge is shown.

1.2 Supplementary Tables

Table S1- Gene product of achromobactin siderophore biosynthesis homologs

Protein	Amino Acid length	Specie	Identity/ Positivies %	Coverage %
**Demethylmenaquinone methyltransferase**	204	*Pseudomonas syringae*	50/71	99
**AcsA**	630	*Pseudomonas syringae*	56/71	98
**AcsB**	255	*Pseudomonas syringae*	66/79	98
**AcsC**	618	*Pseudomonas syringae*	57/72	99
**MFS transporter (YhcA)**	478	*Pseudomonas syringae*	52/70	95
**AcsE (Diaminopimelate decarboxylase)**	407	*Pseudomonas syringae*	61/77	96
**AcsD**	590	*Pseudomonas syringae*	50/65	99
**AcsF (diadenosine tetraphosphatase)**	458	*Pseudomonas chlororaphis*	62/78	94
**Iron ABC transporter substrate-biding protein**	321	Vibrio nereis	70/82	100
		Marinomonas posidonica	63/77	95
		Dickeya zeae	51/79	82
**Iron-siderophore ABC transporter permease**	338	Vibrio nereis	76/87	100
		Marinomonas sp.	65/81	98
		Pseudomnas sp. 655	56/78	89
**Iron-siderophore ABC transporter permease**	344	Vibrio nereis	73/85	99
		Marinomonas posidonica	63/78	99
		Pseudomonas syringae	49/69	99
**Iron-enterobactin transporter ATP-binding protein**	266	*Vibrio nereis*	84/91	96
		Marinomonas sp.	75/84	98
**Ferric iron reductase**	276	*Vibrio nereis*	65/79	96
**Ton-B dependent receptor (Acr)**	668	*Vibrio nereis*	73/84	99
**transposase**	327	*Vibrio crassostreae*	97/96	100

Table S2- Type I restriction modification systems in *V. tapetis*

Cluster number	ID	ID REBASE	Similarity (%)	Organism	Protein ID
**Cluster 1**	Type I restriction-modification system, specificity subunit S (EC 3.1.21.3)	S.Vfl560ORFAP	64	*V. fluvialis*	Q6LR68
	Type I restriction-modification system, DNA-methyltransferase subunit M (EC 2.1.1.72)	M.PprORF1803P	84	*Photobacterium profundum*	CAG20208.1
	hypothetical protein				
	hypothetical protein				
	hypothetical protein				
	Type I restriction-modification system, restriction subunit R (EC 3.1.21.3)	VbaORF1910P	94	*Vibrionales bacterium SWAT-3*	F4LMV8
**Cluster 2**	Putative predicted metal-dependent hydrolase	Vco14ORF11600P	17	*Treponema brennaborense*	A0A097Q
			77	*Vibrio coralliilyticus OCN014*	G2ED23
	hypothetical protein				
	hypothetical protein				A0A097Q
	Type I restriction-modification system, specificity subunit S (EC 3.1.21.3)	S.Bar59ORF2746P	33	*Bizionia argentinensis JUB59*	
	Type I restriction-modification system, DNA-methyltransferase subunit M (EC 2.1.1.72)	M.VspB183ORF2435P			
	Type I restriction-modification system, DNA-methyltransferase subunit M (EC 2.1.1.72)	M2.Vco14ORF11600P			D0KYE4
**Cluster 3**	Type I restriction-modification system, DNA-methyltransferase subunit M (EC 2.1.1.72)	M.VhaBSW7ORF675P	89	*Vibrio harveyi SainMalo-France*	A1SRA3

	Type I restriction-modification system, specificity subunit S (EC 3.1.21.3)	S.Hne2ORF613P	39	*Halothiobacillus neapolitanus c2*	
	Type I restriction-modification system, restriction subunit R (EC 3.1.21.3)	Pin37ORF148P	89	*Psychromonas ingrahamii 37*	
Cluster 4	Type I restriction-modification system, restriction subunit R (EC 3.1.21.3)	VhaE385ORFDP	83	*Vibrio harveyi E385*	
	transposase IS4 family protein				
	Type I restriction-modification system, restriction subunit R (EC 3.1.21.3)	VhaBSW7ORF6735P	94	*Vibrio harveyi BSW7*	A0A0U3R
	Type I restriction-modification system, specificity subunit S (EC 3.1.21.3)	S.AjoXBB1ORF13215P	39	*Acinetobacter johnsonii XBB1*	
	Type I restriction-modification system, DNA-methyltransferase subunit M (EC 2.1.1.72	M.VhaE385ORFDP	93	*Vibrio harveyi E385*	
	Type I restriction-modification system, DNA-methyltransferase subunit M (EC 2.1.1.72)	M.VhaE385ORFDP	44	*Vibrio harveyi E385*	
Cluster 5	Type I restriction-modification system, DNA-methyltransferase subunit M (EC 2.1.1.72)	M.VtaORF26P	100	*Vibrio tapetis*	B2LS99
	Type I restriction-modification system, specificity subunit S (EC 3.1.21.3)	S.VtaORF26P	100	*Vibrio tapetis*	B2LSA0
	Type I restriction-modification system, restriction subunit R (EC 3.1.21.3)	VtaORF26P	100	*Vibrio tapetis*	B2LSA1

Table S3- Number of gene copies related to Type I Restriction Modification systems in *Vibrio tapetis* strains.

Strains	Type I restriction-modification system, specificity subunit S (HsdS)	Type I restriction-modification system, DNA-methyltransferase subunit M (HsdM)	Type I restriction-modification system, restriction subunit R (HsdR)
CECT4600	6	7	6
IS1	6	7	6
IS5	6	7	6
IS7	6	7	6
IS8	6	7	6
IS9	6	7	6
RP2	3	6	6
RP8	6	7	6
RP9	6	7	6
RP11	6	7	6
UK6	6	5	7
P16B	6	7	6
GDE	2	2	2
GTRI	3	2	3
RD0705	6	7	6
HH6087	2	2	2
LP2	2	0	0

Table S4. Upstream and downstream regions of Type IV secretion system cluster of *V. tapetis* CECT4600

CDS	Start	Stop	Size	Function
VTAP_v1_a3482	3640218	3643712	3495	Relaxase TraI (fragment)
VTAP_v1_a3483	3644519	3645382	864	ParB-like partition protein (fragment)
VTAP_v1_a3484	3646263	3646595	333	Toxin HigB-2
VTAP_v1_a3485	3646582	3646896	315	Antitoxin igA-2
VTAP_v1_a3486	3647312	3647803	492	Conserved membrane protein
VTAP_v1_a3487	3648399	3649931	1533	transposase
VTAP_v1_a3488	3649941	3650681	741	conserved protein of unknown function
VTAP_v1_a3489	3650826	3651293	468	conserved protein of unknown function
VTAP_v1_a3490	3651425	3651913	489	conserved protein of unknown function
VTAP_v1_a3491	3652053	3652121	69	protein of unknown function
VTAP_v1_a3492	3652177	3652770	594	transposase
VTAP_v1_a3493	3652958	3653362	405	S-adenosylhomocysteine hydrolase
VTAP_v1_a3494	3653365	3654306	942	conserved protein of unknown function
VTAP_v1_a3495	3654314	3656908	2595	Type I restriction-modification system N6-methylase
VTAP_v1_a3496	3656901	3658160	1260	Type I restriction modification system DNA specificity subunit Hsds
VTAP_v1_a3497	3658322	3661438	3117	Type I restriction modification system deoxyribonuclease subunit HsdR
VTAP_v1_a3498	3662049	3663023	975	putative bacteriophage abortive infection protein
VTAP_v1_a3499	3663249	3663380	132	protein of unknown function
VTAP_v1_a3500	3663391	3663507	117	protein of unknown function
VTAP_v1_a3501	3663704	3663919	216	conserved protein of unknown function
VTAP_v1_a3502	3663879	3664190	312	protein of unknown function
VTAP_v1_a3503	3664187	3664867	681	conserved protein of unknown function
VTAP_v1_a3504	3666230	3666391	162	conserved protein of unknown function
VTAP_v1_a3505	3666388	3666624	237	DinI-like protein MsgA
VTAP_v1_a3506	3666764	3666973	210	conserved protein of unknown function
VTAP_v1_a3507	3667313	3667915	603	conserved protein of unknown function
VTAP_v1_a3508	3667963	3668229	267	protein of unknown function
VTAP_v1_a3509	3668047	3668628	582	transposase
VTAP_v1_a3510	3668761	3668880	120	protein of unknown function
VTAP_v1_a3511	3668976	3669392	417	putative transcriptional regulator
VTAP_v1_a3512	3669401	3669715	315	conserved hypothetical protein
VTAP_v1_a3513	3669915	3670526	612	putative resolvase of Tn3 transposon family
VTAP_v1_a3514	3671303	3672166	864	putative exported nuclease
VTAP_v1_a3515	3672353	3673090	738	transposase
VTAP_v1_a3516	3673660	3673836	177	protein of unknown function
VTAP_v1_a3517	3673778	3673921	144	protein of unknown function
VTAP_v1_a3518	3674420	3674539	120	protein of unknown function
VTAP_v1_a3519	3674836	3674985	150	protein of unknown function
VTAP_v1_a3520	3675820	3676431	612	conserved protein of unknown function
VTAP_v1_a3521	3676446	3676664	219	conserved protein of unknown function
VTAP_v1_a3522	3676897	3677037	141	conserved protein of unknown function
VTAP_v1_a3523	3677325	3677696	372	conserved protein of unknown function
VTAP_v1_a3524	3677762	3677905	144	conserved protein of unknown function
VTAP_v1_a3525	3677898	3678047	150	conserved protein of unknown function
VTAP_v1_a3526	3677996	3678157	162	conserved protein of unknown function
VTAP_v1_a3527	3678144	3678548	405	putative transcription antitermination factor
VTAP_v1_a3528	3678538	3679512	975	ParB-like partition protein

VTAP_v1_a3529	3679509	3680306	798	Partition protein ParA
VTAP_v1_a3530	3681889	3682716	828	putative replication protein
VTAP_v1_a3531	3682703	3682843	141	conserved protein of unknown function
VTAP_v1_a3532	3683212	3683343	132	protein of unknown function
VTAP_v1_a3533	3683490	3683765	276	Resolvase (fragment)
VTAP_v1_a3534	3683934	3684080	147	protein of unknown function
VTAP_v1_a3535	3683995	3685953	1959	putative Methyl-accepting chemotaxis protein
VTAP_v1_a3536	3686334	3686537	204	protein of unknown function
VTAP_v1_a3537	3686771	3687079	309	transposase (fragment)
VTAP_v1_a3538	3687076	3687813	738	transposase
VTAP_v1_a3539	3688006	3689211	1206	protein of unknown function
VTAP_v1_a3540	3689252	3689992	741	conserved protein of unknown function
VTAP_v1_a3541	3690002	3691534	1533	transposase
VTAP_v1_a3542	3691923	3692471	549	TIR protein (fragment)
VTAP_v1_a3543	3692522	3693103	582	conserved protein of unknown function
VTAP_v1_a3544	3693103	3693306	204	conserved protein of unknown function
VTAP_v1_a3545	3693913	3694413	501	protein of unknown function
VTAP_v1_a3546	3694951	3695166	216	protein of unknown function
VTAP_v1_a3547	3695318	3695782	465	transposase
VTAP_v1_a3548	3695851	3697242	1392	transposase
VTAP_v1_a3549	3697392	3697658	267	protein of unknown function
VTAP_v1_a3550	3697779	3697970	192	protein of unknown function
VTAP_v1_a3551	3698089	3698331	243	protein of unknown function
VTAP_v1_a3552	3698379	3698750	372	**putative Type IV secretion system protein B3**
VTAP_v1_a3553	3698743	3699063	321	**putative Type IV secretion system protein B2**
VTAP_v1_a3554	3699066	3701591	2526	**putative Type IV secretion system protein B4**
VTAP_v1_a3555	3701588	3702379	792	**conserved exported protein of unknown function**
VTAP_v1_a3556	3702360	3702584	225	**protein of unknown function**
VTAP_v1_a3557	3702595	3703542	948	**putative Type IV secretion system protein B6**
VTAP_v1_a3558	3703547	3704263	717	**putative Type IV secretion system protein B8**
VTAP_v1_a3559	3704263	3705117	855	**putative Type IV secretion system protein B9**
VTAP_v1_a3560	3705129	3706382	1254	**putative Type IV secretion system protein B10**
VTAP_v1_a3561	3706379	3706732	354	**conserved exported protein of unknown function**
VTAP_v1_a3562	3706729	3707472	744	**putative Type IV secretion system protein D4**
VTAP_v1_a3563	3707473	3708915	1443	**conserved exported protein of unknown function**
VTAP_v1_a3564	3708925	3710046	1122	**conserved protein of unknown function**
VTAP_v1_a3565	3710033	3710779	747	**putative Type IV secretion system protein B11**
VTAP_v1_a3566	3710742	3711974	1233	protein of unknown function
VTAP_v1_a3567	3712060	3712323	264	protein of unknown function
VTAP_v1_a3568	3712453	3712665	213	protein of unknown function
VTAP_v1_a3569	3712741	3713136	396	protein of unknown function
VTAP_v1_a3570	3713150	3713494	345	conserved protein of unknown function
VTAP_v1_a3571	3713511	3713774	264	protein of unknown function
VTAP_v1_a3572	3713883	3714095	213	conserved protein of unknown function
VTAP_v1_a3573	3714076	3714573	498	conserved protein of unknown function
VTAP_v1_a3574	3714830	3715750	921	Antirestriction protein
VTAP_v1_a3575	3715728	3716465	738	transposase
VTAP_v1_a3576	3716844	3717602	759	Transcriptional regulatory protein RstA
VTAP_v1_a3577	3717780	3718022	243	protein of unknown function
VTAP_v1_a3578	3717965	3718285	321	transposase (fragment)
VTAP_v1_a3579	3718484	3718648	165	protein of unknown function
VTAP_v1_a3580	3718621	3718779	159	protein of unknown function
VTAP_v1_a3581	3718656	3718991	336	protein of unknown function
VTAP_v1_a3582	3718991	3719170	180	protein of unknown function
VTAP_v1_a3583	3719461	3720051	591	conserved protein of unknown function
VTAP_v1_a3584	3720048	3720185	138	protein of unknown function
VTAP_v1_a3585	3720291	3720443	153	protein of unknown function
VTAP_v1_a3586	3720460	3722409	1950	Colicin V secretion atp-binding protein
VTAP_v1_a3587	3722406	3723677	1272	Secretion protein
VTAP_v1_a3588	3723668	3725047	1380	Microcin secretion/processing ATP-binding protein

VTAP_v1_a3589	3725113	3725316	204	conserved protein of unknown function
VTAP_v1_a3590	3725362	3725511	150	protein of unknown function
VTAP_v1_a3591	3725593	3725778	186	protein of unknown function
VTAP_v1_a3592	3725784	3726086	303	transposase (fragment)
VTAP_v1_a3593	3726284	3726442	159	protein of unknown function
VTAP_v1_a3594	3726687	3726854	168	conserved protein of unknown function
VTAP_v1_a3595	3726949	3728541	1593	conserved exported protein of unknown function
VTAP_v1_a3596	3728681	3728815	135	protein of unknown function
VTAP_v1_a3597	3728765	3729142	378	transposase
VTAP_v1_a3598	3729250	3729366	117	protein of unknown function
VTAP_v1_a3599	3729544	3730020	477	Insertion sequence IS21 putative ATP-binding protein (fragment)
VTAP_v1_a3600	3730051	3730326	276	transposase (fragment)
VTAP_v1_a3601	3730403	3730720	318	protein of unknown function
VTAP_v1_a3602	3730775	3731512	738	transposase
VTAP_v1_a3603	3731637	3731924	288	Lytic transglycosylase PilT (fragment)
VTAP_v1_a3604	3731986	3732321	336	conserved protein of unknown function
VTAP_v1_a3605	3732406	3732792	387	conserved protein of unknown function
VTAP_v1_a3606	3732816	3733160	345	conserved exported protein of unknown function
VTAP_v1_a3607	3733649	3733840	192	protein of unknown function
VTAP_v1_a3608	3733819	3735642	1824	ParB-like partition protein
VTAP_v1_a3609	3736364	3737548	1185	Abortive infection protein, internal deletion
VTAP_v1_a3610	3737551	3738069	519	conserved protein of unknown function
VTAP_v1_a3611	3738277	3738603	327	conserved protein of unknown function
VTAP_v1_a3612	3738615	3738779	165	protein of unknown function
VTAP_v1_a3613	3738815	3739279	465	conserved protein of unknown function
VTAP_v1_a3614	3739295	3739765	471	conserved protein of unknown function
VTAP_v1_a3615	3740291	3740527	237	DinI-like protein MsgA
VTAP_v1_a3616	3741174	3741776	603	conserved protein of unknown function
VTAP_v1_a3617	3741824	3742789	966	putative transposase
VTAP_v1_a3618	3742825	3742983	159	protein of unknown function
VTAP_v1_a3619	3743205	3743540	336	conserved protein of unknown function
VTAP_v1_a3620	3743588	3744637	1050	Methyl-accepting chemotaxis protein
VTAP_v1_a3621	3744838	3745494	657	Resolvease/recombinase
VTAP_v1_a3622	3745548	3747059	1512	Modification methylase PstI
VTAP_v1_a3623	3747189	3748169	981	Type-2 restriction enzyme PstI
VTAP_v1_a3624	3748581	3751346	2766	conserved protein of unknown function
VTAP_v1_a3625	3751355	3751819	465	conserved protein of unknown function
VTAP_v1_a3626	3752416	3752802	387	conserved protein of unknown function
VTAP_v1_a3627	3752904	3753080	177	conserved protein of unknown function
VTAP_v1_a3628	3753171	3753452	282	conserved protein of unknown function
VTAP_v1_a3629	3753506	3754270	765	ParA family protein
VTAP_v1_a3630	3754304	3754444	141	protein of unknown function
VTAP_v1_a3631	3755580	3756407	828	putative replication protein
VTAP_v1_a3632	3756482	3756592	111	protein of unknown function
VTAP_v1_a3633	3756851	3757495	645	putative HTH-type transcriptional regulator RdgA
VTAP_v1_a3634	3757728	3757862	135	protein of unknown function
VTAP_v1_a3635	3758205	3758693	489	conserved protein of unknown function
VTAP_v1_a3636	3758913	3759200	288	Conjugative transfer protein TraA
VTAP_v1_a3637	3759210	3759512	303	Conjugative protein TraL
VTAP_v1_a3638	3759465	3759653	189	protein of unknown function
VTAP_v1_a3639	3759522	3760097	576	Conjugative pilus assembly protein TraE
VTAP_v1_a3640	3760084	3760875	792	putative conjugative transfer protein TraK
VTAP_v1_a3641	3760865	3761710	846	Conjugativepilus assembly protein TraB (fragment)
VTAP_v1_a3642	3762107	3762340	234	protein of unknown function
VTAP_v1_a3643	3762687	3763379	693	KAP P-loop (fragment)
VTAP_v1_a3644	3763401	3764612	1212	transposase
VTAP_v1_a3645	3764798	3765739	942	KAP P-loop (fragment)
VTAP_v1_a3646	3765739	3766545	807	conserved protein of unknown function
VTAP_v1_a3647	3766545	3767963	1419	conserved protein of unknown function

VTAP_v1_a3648	3767968	3768744	777	TatD-related deoxyribonuclease
VTAP_v1_a3649	3768810	3769265	456	conserved protein of unknown function
VTAP_v1_a3650	3769262	3771277	2016	conserved protein of unknown function
VTAP_v1_a3651	3771277	3773115	1839	conserved protein of unknown function
VTAP_v1_a3652	3773112	3774692	1581	conserved protein of unknown function
VTAP_v1_a3653	3774825	3775337	513	Regulator of ribonuclease activity B
VTAP_v1_a3654	3775816	3777036	1221	Arginine deiminase
VTAP_v1_a3655	3777190	3778194	1005	ornithine carbamoyltransferase 1
VTAP_v1_a3656	3778421	3779350	930	aspartate carbamoyltransferase, catalytic subunit
VTAP_v1_a3657	3779367	3779828	462	aspartate carbamoyltransferase, regulatory subunit
VTAP_v1_a3658	3780415	3781212	798	putative ABC-type metal ion transport system, periplasmic component/surface adhesin
VTAP_v1_a3659	3781308	3782021	714	putative ABC-type transport system, ATPase component
VTAP_v1_a3660	3782018	3783277	1260	putative ABC-type antimicrobial peptide transport system, permease component
VTAP_v1_a3661	3783290	3783790	501	conserved exported protein of unknown function
VTAP_v1_a3662	3784102	3785001	900	conserved protein of unknown function
VTAP_v1_a3663	3785173	3786456	1284	conserved protein of unknown function
VTAP_v1_a3664	3787269	3788348	1080	protein chain elongation factor EF-Tu, possible GTP-binding factor (duplicate of tufA)
